# Pathogens Manipulating Tick Behavior—Through a Glass, Darkly

**DOI:** 10.3390/pathogens9080664

**Published:** 2020-08-17

**Authors:** Giovanni Benelli

**Affiliations:** Department of Agriculture, Food and Environment, University of Pisa, via del Borghetto 80, 56124 Pisa, Italy; giovanni.benelli@unipi.it; Tel.: +39-050-221-6141

**Keywords:** *Anaplasma*, *Babesia*, *Bartonella*, *Borrelia*, tick ecology and evolution, Lyme disease, host seeking, *Ixodes*, questing, *Rickettsia*, tick-borne encephalitis virus, tick management

## Abstract

Pathogens can manipulate the phenotypic traits of their hosts and vectors, maximizing their own fitness. Among the phenotypic traits that can be modified, manipulating vector behavior represents one of the most fascinating facets. How pathogens infection affects behavioral traits of key insect vectors has been extensively investigated. Major examples include *Plasmodium*, *Leishmania* and *Trypanosoma* spp. manipulating the behavior of mosquitoes, sand flies and kissing bugs, respectively. However, research on how pathogens can modify tick behavior is patchy. This review focuses on current knowledge about the behavioral changes triggered by *Anaplasma*, *Borrelia*, *Babesia*, *Bartonella*, *Rickettsia* and tick-borne encephalitis virus (TBEV) infection in tick vectors, analyzing their potential adaptive significance. As a general trend, being infected by *Borrelia* and TBEV boosts tick mobility (both questing and walking activity). *Borrelia* and *Anaplasma* infection magnifies *Ixodes* desiccation resistance, triggering physiological changes (*Borrelia*: higher fat reserves; *Anaplasma*: synthesis of heat shock proteins). *Anaplasma* infection also improves cold resistance in infected ticks through synthesis of an antifreeze glycoprotein. Being infected by *Anaplasma*, *Borrelia* and *Babesia* leads to increased tick survival. *Borrelia*, *Babesia* and *Bartonella* infection facilitates blood engorgement. In the last section, current challenges for future studies are outlined.

## 1. Introduction

Vector-borne diseases (VBDs) are caused by parasites, bacteria and viruses, leading to more than 700,000 deaths yearly [1]. Many VBDs are caused by pathogens vectored by arthropods, among which mosquitoes, sand flies, triatomine bugs and ticks are major players [2,3,4]. Pathogens represent a significant selective pressure on their hosts [5]. The pathogen-host interaction shapes coevolution on both sides. Pathogens can manipulate many phenotypic traits of their hosts, thus maximizing their fitness [6,7,8]. A classic example is represented by *Plasmodium* infection making host-borne odors more attractive to *Anopheles* mosquitoes [9,10,11] (but see [12]). Similarly, *Leishmania* modify the odor of infected hosts, making it more attractive for sand flies [13,14], and *Hepatozoon* infection can lead to odor changes in snake and frog hosts, which leads to higher feeding rates by *Culex pipiens* and *Culex territans* mosquito vectors [15].

Among the phenotypic traits that can be modified, a fascinating category is represented by the behavioral manipulation of vectors to enhance pathogens transmission. According to an earlier classification by Hurd [16], manipulation can be achieved through various mechanisms, such as boosting the chances of contacts among vectors and hosts, reducing vector reproduction to increase nutrients available for the microorganisms, and/or magnifying vector survival.

Behavioral alterations caused by pathogens on their vectors include higher biting rates, reported for *Yersinia pestis*-infected *Xenopsylla cheopis* fleas [17], La Crosse virus-infected *Aedes triseriatus* mosquitoes [18,19], *Trypanosoma cruzi*-infected *Mepraia spinolai* kissing bugs [20], and *Leishmania mexicana*-infected *Lutzomyia longipalpis* sandflies [21], to cite some key examples. Furthermore, pathogen infection can lead to longer biting duration, as observed for dengue-infected *Aedesa egypti* [22], *Trypanosoma brucei*- and *Trypanosoma congolense*-infected *Glossina morsitans morsitans* [23,24], *Trypanosoma rangeli*-infected *Rhodnius prolixus* [25], and *Leishmania major*-infected *Phlebotomus duboscqi* [26]. Increased host searching ability is another possible consequence of the pathogen infection, as recently outlined for young instars of *Triatoma pallidipennis* and *Triatoma longipennis* infected by *Trypanosoma cruzi*, which are more active and able to detect the human odor than non-infected individuals [27]. Moreover, dengue-infected *Ae*. *aegypti* mosquitoes show an overall increase of their locomotor ability [28], which can boost their likelihood to detect potential hosts. Lastly, pathogen infection can improve the mating performances of a given arthropod vector, as reported for La Crosse virus-infected *Ae*. *triseriatus*; infected mosquitoes mate earlier than non-infected ones [29]. Of note, pathogens-induced behavioral changes in their vectors have been extensively studied by malaria researchers. Higher biting rates and/or longer biting duration have been reported for several *Anopheles* species infected by *Plasmodium* spp. [6,30,31,32,33], in some cases coupled with increased survival [34].

Examples of pathogens promoting longer vector lifespan are sparser than behavioral changes, being reported for *T*. *brucei gambiense*-infected *Glossina palpalis* [35] and *T. brucei rhodesiense*- and *T*. *brucei*-infected *G*. *morsitans morsitans* [36,37]. In contrast, pathogen infection can also lead to reduced lifespan and fecundity, as reported for *Leishmania*-infected sandflies [38], *Cx. pipiens* infected by the Rift Valley fever virus [39], and *Trypanosoma*-infected triatomine bugs [40,41,42], as examples of possible evolutionary arms races [27].

It has been stressed that manipulation of vector phenotypic traits is more widespread among parasites characterized by a complex life cycle. The latter require a longer and complicated series of events to complete the life cycle, which can be completed more easily through host manipulation [27,43,44]. This scenario may also fit ticks vectoring pathogens responsible of tick-borne diseases. A key example is the complex life cycles of *Ixodes ricinus* and *Ixodes scapularis* on multiple hosts over time, which are closely connected with the spread of Lyme borreliosis [45]. However, if compared to the behavioral changes widely investigated in the pathogen-insect vector interactions mentioned above, our knowledge about how pathogens can modify the behavior of tick vectors is patchy, and an overall analysis of the literature available about all major tick-borne pathogens potentially impacting tick behavior is still lacking. In particular, Lyme disease is on the rise [46,47], and a full understanding of how the interaction between its causative agent *Borrelia* spp. and the tick vectors can lead to behavioral modifications in the latter is of utmost value. The present review focuses on current knowledge on the behavioral changes triggered by *Anaplasma*, *Borrelia*, *Babesia*, *Bartonella*, *Rickettsia* and tick-borne encephalitis virus (TBEV) infection in tick vectors, with a focus on their potential adaptive significance and related fitness implications.

## 2. Pathogens Infecting Ticks Lead to Major Behavioral Changes

Most of the studies about how pathogen infection can affect tick behavior have focused on *Borrelia* bacteria, the causative agents of Lyme borreliosis, followed by *Anaplasma*-related studies. Only a few studies have considered the behavioral changes triggered by *Babesia*, *Bartonella*, *Rickettsia*, and TBEV infection. The following subsections review current knowledge about how infection with different pathogens may affect tick behavior. The first subsection is dedicated to the behavioral changes in *Borrelia*-infected ticks, followed by subsections focusing on the behavioral changes in ticks infected by other pathogens. Since it is hard to disentangle behavioral results from their whole biological context, this review critically discusses behavioral research along with current information on the impact of pathogen infection on other phenotypic traits. Physiological research shedding light on how pathogens can finely manipulate tick basic metabolism shows how this profoundly impacts tick behavior and ecology.

### 2.1. Behavioral Changes in Borrelia-Infected Ticks

#### 2.1.1. Laboratory Studies

Several studies demonstrate how *Borrelia* spp. can promote behavioral modifications in tick vectors. A pioneer study by Lefcort and Durden [48] highlighted that nymphs of *I. scapularis*, infected by *Borrelia burgdorferi*, showed increased phototaxis and attraction to vertical surfaces compared to non-infected ticks. Both behavioral alterations may be adaptive, boosting *Borrelia* transmission, since increased phototaxis and attraction to vertical surfaces could increase the likelihood of contact between the tick vector and a potential reservoir host. Additionally, from a physiological point of view, it has been reported that *B*. *burgdorferi*-infected *I*. *scapularis* ticks can up-regulate the tick histamine release factor (tHRF), allowing a higher blood flow to the bite site and improved vascular permeability. In this way, ticks may engorge more quickly, and the likelihood of becoming infected by *Borrelia* is also higher [49]. In contrast, *B. burgdorferi*-infected *I*. *scapularis* adults showed lower mobility than uninfected ones, questing at lower heights, being less able to overcome physical obstacles, and avoiding climbing on vertical surfaces [48]. Reduced mobility of adult ticks may represent a by-product of *B*. *burgdorferi* infection, possibly non-adaptive for the pathogen or for the tick. However, one may consider that this behavior may be adaptive, since reduced tick movement contribute reducing water and energy loss. Current evidence does not allow any definitive conclusion. While *I*. *scapularis* nymphs are crucial for infecting reservoir hosts, thus indirectly newly hatched tick larvae, adults are less prone to feed on reservoir hosts, and so they are not considered mayor players in the spread of *Borrelia* bacteria [45]. However, these conclusions cannot be generalized for all *Ixodes* species. Indeed, further research by Romashchenko et al. [50] showed that *I*. *persulcatus* adults with a higher prevalence of *B*. *burgdorferi* s.l. reached a higher questing height (where the likelihood of intercepting a large host for feeding purposes is higher) than non-infected ticks.

Larvae, nymphs and adults of *I. ricinus* naturally infected by *B. burgdorferi* s.l. showed reduced mobility compared to uninfected ones [51]. The same applies to *Borrelia*-infected adults of the taiga tick, *I. persulcatus* [51,52,53], suggesting that being infected by *B. burgdorferi* s.l. boosts mobility of *I*. *persulcatus* adults with exoskeleton deformities (i.e., crater-like depressions on the exoskeleton, damaged female scutum and undeveloped palps), collected from a heavy metal polluted area. The reasons at the basis of the observed differences are unclear, and may be partially linked to the short observation time of the mobility experiments (i.e., 3 min). Later, at variance with the results by Alekseev and co-workers, Perret [54] reported that *I. ricinus* nymphs naturally infected by *B. burgdorferi* s.l. exposed to desiccating conditions for a prolonged time are more active than uninfected ones. Similarly, *Borrelia afzelii*-infected *I. ricinus* nymphs showed higher mobility [i.e., longer walking activity and higher velocity (+10%)] over uninfected ones [55].

#### 2.1.2. Into the Woods—Field Studies

In addition to laboratory research, it is important to consider what is known about the impact of *Borrelia* infection on ticks in the natural setting. A flagging collection study in the proximity of St. Petersburg (Russia) [56] showed that the appearance of *B*. *burgdorferi* s.l.-infected *I*. *persulcatus* ticks was related to gradients between the surface and soil temperature and the soil and air relative humidity. More *Borrelia*-infected nymphs were detected at the temperature interval 10–14 °C compared to 15–20 °C and 21–26 °C (no collected individuals in this latter case) [56]. In dense woodlands nymphs of the western black-legged tick, *Ixodes pacificus*, infected by *B*. *burgdorferi* were found at higher densities on logs and trunks than in the leaf litter [57]. A field study carried out on *I*. *ricinus* in western Germany showed that *B*. *burgdorferi* s.l.-infected adult females were more frequently present on protective clothes of human volunteers compared to infected females caught by blanket dragging in the same study area [58]. These field results highlight that being infected by *Borrelia* may contribute to boost host-finding strategies at least for *I*. *pacificus* and *I*. *ricinus*. Further field data on other *Ixodes* species are needed. It is also important to determine the effects of other *Borrelia* genospecies on tick behavioral traits.

#### 2.1.3. *Borrelia* Manipulation of the Host Odors

Do *Borrelia* infections manipulate host odor that make hosts more attractive for tick vectors? Similar questions have been addressed about other pathogens, making the host odor more attractive for their insect vectors [13,59]. However, for ticks this question remained unanswered. Recently, van Duijvendijk et al. [60] showed that *I*. *ricinus* nymphs were more attracted to bank voles (*Myodes glareolus*) infected by *B. afzelii* over uninfected voles, and that infected nymphs feeding on bank voles showed a higher body weight than uninfected ones.

But what happens when the tick vector selects a host not competent for *Borrelia* transmission? Is the pathogen able to manipulate the tick vector boosting avoidance of the incompetent host? Berret and Voordouw [61] attempted a reply to both questions, showing that being infected by rodent- or bird-specialized *Borrelia* genospecies did not affect attraction (in terms of questing rates)to mouse odor. Thus, no evidence for the qualitative manipulation hypothesis [6] in the *Borrelia*-*Ixodes* interaction has been detected.

#### 2.1.4. *Ixodes* Behavior Meets Physiology

*Borrelia* affects tick physiology in several ways. For example, *B. burgdorferi* infecting *I*. *scapularis* salivary glands can usurp a tick salivary protein, *salp15*, which binds to the spirochetes protecting them from antibody-mediated killing; thus, facilitating infection in mice [62]. The studies summarized above (with few exceptions, see [51,52,53]) highlight that *Borrelia* infection–as a general trend–impacts tick mobility, with special reference to host-seeking activity. One may consider that a more mobile tick is exposed to a higher risk of desiccation (thus needing to move more often in the ground litter to recover water), and may also need higher energy reserves, because of its increased metabolic activity [63]. The study by Herrmann and Gern [64] shows that the infection by *B*. *burgdorferi* s.l. makes *I*. *ricinus* ticks more resistant to desiccation when exposed to challenging thermo-hygrometric conditions. The effect was more pronounced on female ticks than on males and nymphs, and highest survival was noted for *B*. *afzelii*-infected ticks [64]. A further study examined *I*. *ricinus* nymphs collected in the field, showing that *Borrelia*-infected ones lived longer than uninfected conspecifics when exposed to unfavorable thermo-hygrometric parameters [55].

As concerns the impact of *Borrelia* infection on tick energy reserves, it has been reported that *I*. *ricinus* nymphs questing in the field have a higher fat content when infected by *B*. *burgdorferi* s.l. if compared to uninfected ones [65]. The same study clarified that *Borrelia* exploited only a tiny fraction of these energy reserves, thus the tick can benefit from the increased fat reserves for its own metabolism [65]. As stressed by Herrmann and Gern [63], ticks water recovering occurs through active absorption of external aqueous vapor, and this process is costly from an energetic point of view. Thus, the higher energy reserves of *Borrelia*-infected ticks can contribute to allow a higher resistance to desiccation.

*Borrelia burgdorferi* s.l. infection and energy reserves affect horizontal mobility in *I*. *ricinus* nymphs. Ticks with high-fat reserves are more prone to explore dryer areas, but—among them—infected individuals stay longer in a fixed position [66]. This partially contrasts with the earlier findings by Lefcort and Durden [48]. Anyway, it has been claimed that *Borrelia* manipulates *I. Ricinus* nymphs to remain still, waiting for possible hosts, even under desiccating conditions that usually increase walking activity in non-infected conspecifics [66]. The overall picture is interesting and outlines how *Borrelia* infection contribute in multiple ways to its own and vector fitness. Remaining still under challenging thermo-hygrometric conditions is highly dangerous for ticks, and only individuals with abundant energy reserves can perform a prolonged recovery of water vapor from the air [67]. The adaptive significance of this behavior deserves further studies, as well as validation of the findings on other *Ixodes* species.

Lastly, understanding how the behavioral changes characterizing *Borrelia*-infected ticks may contribute to the spread of Lyme disease is a crucial, but neglected, research topic. A theoretical model developed by Gassner and Hartemink [45] stresses how behavioral changes triggered by *Borrelia* infection may contribute to an increased transmission risk. Applying a next-generation matrix approach the authors showed that the increased tick vector survival deeply affects the basic reproduction number R_0_ for *Borrelia* pathogens.

### 2.2. Behavioral Changes in Anaplasma-Infected Ticks

*Anaplasma* infection leads to important physiological changes in *Ixodes* ticks, impacting tick survival, questing, and feeding activity [68,69,70]. Indeed, as recently highlighted by Cabezas-Cruz et al. [71], *Anaplasma phagocytophilum* infecting *I*. *scapularis* manipulates the expression of key genes, boosting in turn both vector fitness and the pathogen transmission [68,72]. Three major facts related to *Anaplasma* infection on ticks deserve consideration.

First, ticks exposed to challenging thermo-hygrometric conditions experience a high risk of desiccation. *Anaplasma phagocytophilum* infecting *I*. *scapularis* ticks induce the synthesis of heat shock proteins (i.e., *hsp20* and *hsp70*), which reduce the risk of desiccation, thus enhancing tick survival rates [73]. From a behavioral point of view, gene knock-down of these *hsp* is linked with reduced questing speed in *I*. *scapularis* males, but with relevant changes according to the tested temperature. At 22 °C, only the knock-down of *hsp70* gene leads to a significant decrease in tick questing activity (−50%) [73]. *Anaplasma phagocytophilum* infection does not downregulate the *I*. *scapularis* production of the tick protective antigen subolesin, which–if knocked down artificially—leads to reduced questing rates (i.e., −50% and −66% at 4 °C and 37 °C, respectively) [73], among other negative effects on tick biology [71]. The production of another crucial protein for vectors of *A*. *phagocytophilum*, such as *I*. *scapularis* and *I*. *ricinus*, the tudor staphylococcal nuclease, is not affected by the pathogen, thus minimizing damage for tick feeding (assessed in term of tick weight), thus their survival [74].

Second, being exposed to cold temperatures during the winter may lead to significantly higher mortality rates in tick populations. *Anaplasma* infection can lead to beneficial effects on its tick vectors. Indeed, it has been showed that the infection of *I*. *scapularis* nymphs by *A. phagocytophilum* trigger the synthesis of an antifreeze glycoprotein that protect ticks from cold [75].

Third, *A*. *phagocytophilum* infecting the salivary glands of *I*. *scapularis* facilitates the infection process through inhibition of the intrinsic apoptosis pathway, while tick cells respond through the extrinsic apoptosis pathway limiting the infection and boosting vector feeding and survival [69,71,76,77].

Overall, the interaction between *A*. *phagocytophilum-I. scapularis* is one of the better studied pathogen-tick relationships, with fully clarified mechanisms magnifying tick and pathogen fitness. Applying this successful approach for other important *Anaplasma* species (e.g., *A*. *marginale*, *A*. *centrale*, *A*. *ovis* and *A*. *platys*) and their tick vectors is an important timely challenge.

### 2.3. Behavioral Changes in Babesia-Infected Ticks

At variance with the plethora of studies on the effect of *Borrelia* infection on tick behavior, the impact of *Babesia* infection on tick vectors has been considered only in a couple of studies. First, Randolph [78] showed that *Babesia microti* infection magnified the feeding success (in terms of mean engorged weight) and survival (in terms of the percentage molt of larvae to nymphs) of the shrew tick *Ixodes trianguliceps*. Both changes are not linked to the level of infection by *B*. *microti* [78]. Later, Hu et al. [79] focused on the potential effect of *B*. *microti* infection on *I*. *scapularis* feeding time, engorged body weight, and molting rate. Being infected delays the tick engorgement, but the body weight of nymphs engorged on infected hosts is higher than that of the conspecifics that fed on uninfected ones. Larvae, but not nymphs, fed on infected hosts show higher molting rates than those that fed on non-infected hamsters. These differences are not detected testing various tick instars on a different host, i.e., *Peromyscus leucopus* mice [79]. The mechanisms that promote feeding success, development and survival of *Babesia*-infected ticks are unknown.

### 2.4. Behavioral Changes in Bartonella-Infected Ticks

Studies on the behavioral effects of *Bartonella* infection in ticks are limited. Recently, the salivary glands transcriptome of *I*. *ricinus* females infected or not by *Bartonella henselae* was investigated. *Bartonella henselae* infection of salivary glands up-regulate *I. ricinus* serine protease inhibitor (IrSPI), a member of the BPTI/Kunitz family of serine protease inhibitors, and IrSPI silencing strongly reduces weight of *I*. *ricinus* feeding adults [80].

### 2.5. Behavioral Changes in Rickettsia-Infected Ticks

Despite the public health importance of rickettsiosis vectored by ticks, our knowledge about how the *Rickettsia* infection may affect the behavior of tick vectors is limited. Frątczak et al. [81] showed that *I*. *ricinus* adult males and females are attracted toward an area irradiated by a 900 MHz electromagnetic field in a radiation-shielded tube. Ticks infected by *Rickettsia* spp. as well as those co-infected by *Rickettsia* spp. and *Borrelia* s.l. are more attracted by the electromagnetic field compared to the uninfected ones [81]. Understanding the effects of anthropogenic environmental modifications on the pathogen-tick interaction and the related changes in vector behavior represents a highly valuable area of research, which is of practical interest for public health.

### 2.6. Behavioral Changes in TBEV-Infected Ticks

Ixodid ticks feeding on humans or animals show a higher TBEV prevalence compared to field-collected ticks from the same area. This is clear with *I*. *ricinus* from Germany [82] as well *I*. *persulcatus* and *Ixodes pavlovskyi* from Russia [83,84]. Two main hypotheses have been formulated to explain these findings. First, the TBEV infection magnifies tick mobility and host-seeking activity. Second, feeding boosts the titer of TBEV already present in unfed questing ticks to a detectable level. The study by Belova et al. [85] provided data in support of both theories. *Ixodes ricinus* adults infected by TBEV trying to reach a bait are more active and tolerant than non-infected ones when exposed to growing concentrations of the repellent N,N-diethyl-meta-toluamide (DEET). Of note, about 6% of the infected adults can go over an area where DEET was formulated at 1%, while none of the uninfected ticks could do so [85].

Furthermore, it should be noted that TBEV-infected *I*. *persulcatus* females are more active, showing a higher mobility both in terms of walking speed and length of the trajectory when trying to reach a bait [86]. Moreover, *I. persulcatus* infected with the TBEV reach a higher questing height compared to uninfected conspecifics [50,53].

## 3. Concluding Remarks and Outstanding Challenges for a Research Agenda

This analysis of current knowledge about how, when and why pathogens trigger behavioral changes in their tick vectors shows that most studies focused on *Ixodes* species infected by *Borrelia* bacteria, followed by research on *Anaplasma*-*Ixodes* interactions. Only a limited number of studies investigated tick behavioral changes associated to infection by *Babesia*, *Bartonella*, *Rickettsia*, and TBEV.

**Figure 1:**
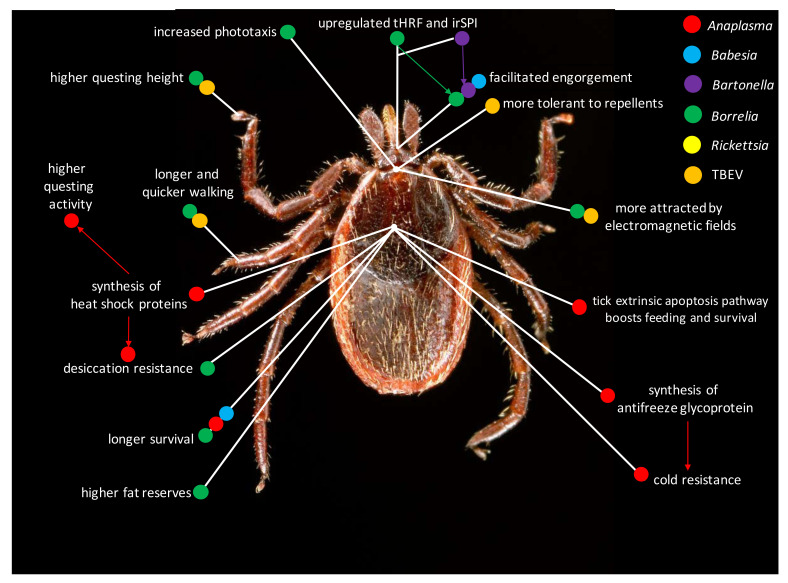
Main behavioral changes caused by pathogen infection of the tick vector. Colored dots indicate different pathogens; pathogen-triggered key physiological changes contributing to increase tick feeding, survival or the vector, and likelihood of coming in contacts with potential hosts are also outlined; TBEV: tick-borne encephalitis virus; tHRF: tick histamine release factor; IrSPI: *I. ricinus* serine protease inhibitor.

However, even if limited, the knowledge available on the topic contributes to depict a fascinating scenario. As a general trend, being infected by *Borrelia* and TBEV substantially increase the overall tick mobility, with special reference to exploiting higher questing heights (this is likely connected with increased phototaxis), often coupled with a higher walking speed and longer walking activity, if compared to non-infected ticks. Noteworthy analogies have been found examining changes triggered post-*Borrelia* and *Anaplasma* infection. Both pathogens magnify desiccation resistance of *Ixodes* ticks (Figure 1). This has been linked to fine physiological changes, i.e., storing higher fat reserves in *Borrelia*-infected ticks, and synthesizing heat shock proteins in *Anaplasma*-infected ticks. *Anaplasma* also improves cold resistance in infected ticks. Additionally, being infected by both pathogens, as well as by *B*. *microti*, allowed ticks to achieve a longer survival. *Babesia* infection also led to higher feeding success (i.e., higher engorged weight), a successful engorgement through upregulation of tHRF and IrSPI characterizes *Borrelia*- and *Bartonella*-infected ticks, respectively (Figure 1). Overall, behavioral and physiological changes triggered by pathogens substantially contribute to the tick fitness [63,71], fostering a win-win strategy in the pathogen-tick interaction [69].

Following the criteria expressed for parasite/pathogen-host interactions [7], the term “manipulation” can be used only when the research shows direct fitness benefits (thus an adaptive value of the observed change) for the infecting pathogen, mainly improving its transmission and dispersal rates. This is not disconnected from benefits for the tick vector, often including a longer survival (Figure 1). In other cases, reducing the infection (as in the *A*. *phagocytophilum*-*Ixodes* interaction) or lowering its detrimental effects, can also lead to significant changes, which can be beneficial for the vector. Even the pathological state triggered by the infection can lead to behavioral changes [7,87], which may even reduce the vectorial ability of the involved species. These seem the case of earlier studies showing reduced feeding rates in *Cx. pipiens* infected by the Rift Valley fever virus [39], as well as the decreased flight activity in *Cx. tarsalis* infected by the Western equine encephalomyelitis virus [88]. Other pathogen-vector interactions need to be studied further, e.g., the vesicular stomatitis virus-*Culicoides sonorensis* one, which led to a decrease in the midge biting behavior 2 days’ post-inoculation (DPI), followed by a sharp increase 3 DPI, but not 4 DPI [89]. Besides these examples on insects, a possible case fitting this category can be that of *B. burgdorferi*-infecting *I*. *scapularis* adults, which results in lower tick mobility, with detrimental effects also on questing [48]. Overall, caution remains the first and most useful rule when using the term “adaptive”.

In conclusion, based on the analysis of the literature considered in this review, there are some points that can be useful for developing a perspective research agenda.

Despite the wide diversity of tick species worldwide [90], only *Ixodes* species have been considered in studies analyzing pathogen-related behavioral changes. How pathogens affect behavioral traits of other tick vectors of huge medical importance, e.g., those belonging to the genera *Amblyomma*, *Dermacentor*, and *Rhipicephalus*, is still overlooked.

Most of the studies have been carried out on *Anaplasma*- and *Borrelia*-*Ixodes* interactions; more research efforts on other key tick-borne pathogens (with special reference to *Babesia*, *Coxiella*, *Ehrlichia*, *Rickettsia*, and *Theileria* species) are urgently needed, elucidating the physiological pathways promoting vector manipulation, as recently carried out for *Anaplasma* and *Bartonella* research [69,71,80].

Standardizing methods for behavioral research is crucial to allow data comparisons. For example, high reproducibility of questing-related data can be obtained using mechatronic arenas, which also reduce animal testing in laboratory [91], and allows the dissection of host-borne cues [92]. The use of mechatronic arenas also allows repeated testing, thus assessing both intra- and inter-individual variability (e.g., questing success and responses to repellents) of the affected traits in highly standardized conditions over time.

While most studies focused on the effect of pathogen infection on mobility, questing and feeding activities, one may consider, they affect the timing of mating (as showed for *Ae*. *triseriatus* infected by La Crosse virus, [29]), mate recognition and mating success of ticks? To the best of my knowledge, this issue has never been investigated.

Moving the focus from studying single traits to a more systematic analysis of all behavioral traits potentially altered by the pathogen infection, and how they correlate each other, represents a major challenge for future research [7].

Both for *Borrelia* and TBEV-infected ticks, field data have been found extremely useful, fitting the behavioral changes observed in laboratory, which may be partially responsible of enhanced host-seeking activities. Further field research focusing on the impact of other pathogen infections on tick behavioral and ecological traits is needed.

Lastly, the role of manipulation is still not considered in most epidemiological models [10], and the behavioral differences between infected and uninfected vectors need to be carefully evaluated for the correct development and implementation of tick control tools within the Integrated Vector Management and One Heath approaches [2,93,94].
